# Time-frequency analysis and autoencoder approach for network traffic anomaly detection

**DOI:** 10.1016/j.mex.2025.103228

**Published:** 2025-02-17

**Authors:** Ruchira Purohit, Satish Kumar, Sameer Sayyad, Ketan Kotecha

**Affiliations:** a Symbiosis Institute of Technology, Symbiosis International (Deemed University), Pune, Maharashtra, India; b Symbiosis Centre for Applied Artificial Intelligence, Symbiosis International (Deemed University), Pune, Maharashtra, India; c Peoples’ Friendship University of Russia named after Patrice Lumumba (RUDN University), Miklukho-Maklaya Str. 6, Moscow, 117198, Russian Federation

**Keywords:** Anomaly detection, Autoencoders, Continuous wavelet transform, Discrete-time Fourier transform, Hybrid time-frequency analysis, Network traffic, Short-time Fourier transform, Hybrid Time-Frequency Analysis and Autoencoder

## Abstract

Detection of anomalies in network traffic is critical to mitigating cyber threats. This study integrates continuous wavelet transform (CWT), discrete-time Fourier transform (DTFT), short-time Fourier transform (STFT), and autoencoders to identify anomalous network behaviour. It conducts time- frequency analysis of pre-processed network traffic data such as packet size and duration, extracting meaningful features fed into an autoencoder. Reconstruction error deviations indicate anomalies like spikes or irregular oscillations.

• This hybrid approach demonstrates good scalability for the real-time implementation of cybersecurity measures. Further developments can be made in autoencoder architectures to achieve their full potential in large-scale systems.
• The model is robust and scalable for real-time applications, achieving 95% detection accuracy by identifying 72 anomalies.
• Obtained results indicate that the approach is feasible for deploying in practical cybersecurity applications.

## Graphical abstract

**Figure:**
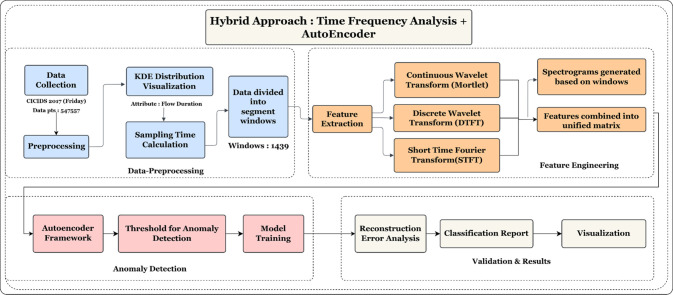

Specifications table

**Table:** 

Subject area:	Computer Science
More specific subject area:	Network Traffic Anomaly Detection
Name of your method:	Hybrid Time-Frequency Analysis and Autoencoder
Name and reference of original method:	Shon, Taeshik, and Jongsub Moon. “A hybrid machine learning approach to network anomaly detection.” Information Sciences 177, no. 18 (2007): 3799–3821.
Resource availability:	Data: Data available on request

## Background

In the digital world today, the security of network systems is more important than ever. The rapid growth in internet-based services is creating vulnerabilities in network infrastructures to a wide variety of cyberattacks, including Distributed Denial of Service (DDoS), phishing, malware, and other forms of intrusion. Therefore, detection of such attacks as early as possible can prevent data breaches, financial losses, and system compromises. Traditional network intrusion detection techniques mainly depend on signature-based or rule-based approaches, which often miss the new and unknown patterns of attacks. As such, there is growing interest in using machine learning and advanced signal processing to find anomalies in network traffic and improve the robustness of intrusion detection systems. One such approach is time-frequency analysis techniques, which provide a robust framework for analyzing signals both in the time and the frequency domains. Such approaches are especially effective in bringing out hidden patterns in complex, dynamic network traffic data. Three popular time-frequency transforms that are suitable for network traffic analysis because they can display both short-term and long-term frequency variations are the Continuous Wavelet Transform (CWT), the Discrete-Time Fourier Transform (DTFT), and the Short-Time Fourier Transform (STFT) [1].

The Continuous Wavelet Transform has a wide range of applications due to its capability of multi-resolution analysis and capturing transient features that may occur at various scales [2]. Adjusting the wavelet scale provides for an elaborate view in terms of how signal frequency content changes over time so that minute anomalies may be sensed as having potential security issues. In contrast, DTFT will show a frequency-domain representation of a signal useful in picking up periodic behavior and possibly abnormal spikes in changes to the traffic pattern [3]. The STFT is another kind of time-frequency analysis tool. Here, the signal is segmented into smaller overlapping sections that undergo Fourier analysis. So, both time and frequency characteristics of the signal can be captured [4].

Although these time-frequency analysis tools are very efficient at capturing anomalies in network traffic, they do not, of course, inherently learn the new patterns of network behavior or improve over time. This limitation can be overcome in the anomaly detection framework by adding machine learning techniques, such as autoencoders. Autoencoders represent a kind of neural network that learns unsupervised to compress data and try to reconstruct it in an autoencoder manner. In the process of normal network traffic pattern training in the autoencoder, model reconstruction for normal network patterns is highly accurate. Any anomalous traffic will provoke an increase in reconstruction errors, thus signalling the presence of an anomaly [5,6].

In this work, we propose a hybrid approach that combines time-frequency analysis techniques, namely CWT, DTFT, and STFT, with autoencoders for enhanced network traffic anomaly detection. This integration of both classical signal processing methods and modern machine learning techniques allows the development of a more robust, adaptive, and scalable solution for network security. It intends to prove how the introduction of time-frequency analysis together with autoencoders makes for a substantial improvement in discovering subtle anomalies in network traffic that otherwise would have bypassed standard methods [7,8].

Network traffic anomaly detection is an important function in ensuring the security and integrity of modern networks, especially due to increasing threats and complexity within network traffic. Researchers utilized various methods that combined older statistical methodologies, wavelet analyses, and machine learning strategies to confront the multi-domain nature of anomaly detection. Identification of network traffic anomalies is one of the most critical issues that both administrators and researchers deal with nowadays; hence, timeliness in discovering these abnormalities would save serious consequences. Therefore, in this experiment [9], we test the idea that can relate the interval-based features of network traffic with many kinds of anomalies. For that, we implemented two well-known algorithms of machine learning: Naive Bayes and K-Nearest Neighbors (KNN) for the purpose of testing their ability to classify and to better understand anomalies in general. With such insight on the feature selection for anomalies, these researchers and administrators of a network may choose the effective interval-based feature that is proper for specific anomalies and select even the correct machine learning algorithm that will help in optimizing its anomaly detection efficiency as well as the accuracy on the network system being utilized. This is then furthered with the concept of a hybrid framework between DBN and long short- term memory, which fully takes advantage of the existing time dependencies within network traffic data. Their approach capitalized on the generative capability of DBNs for pretrained initializations of LSTM networks in order to improve better modeling of sequential patterns by enhancing the model's capabilities over time for anomaly detection. This is considered a significant advancement in how deep learning techniques can be combined to address the dynamic nature of network traffic, therefore offering higher accuracy in the detection of anomalies than conventional models [10]. Further investigation into wavelet analysis application was pursued as Analytical Discrete Wavelet Transforms were exploited to decompose traffic signals onto different frequency bands. Decomposing the signal at very granular levels constitutes a basis for the discovery of network anomalies with little-to-no false positives in live deployments [11]. The integration of Principal Component Analysis with wavelet transforms was introduced in [12], which shows that methods of dimensionality reduction could complement wavelet-based anomaly detection. PCA helps focus computational resources on the most informative features by reducing complexity in high-dimensional network traffic data, thus making the wavelet-based models efficient and scalable. It's particularly valuable in environments where computational efficiency is critical, like real-time anomaly detection in large-scale networks. Expanded on multi-scale anomaly detection techniques through adaptations of wavelet transforms for high-speed network environments. Their research highlights the need for scalable anomaly detection models, considering that the increasing rate of traffic in networks requires a process that can handle huge data volumes without compromising on the accuracy of the outcome [13]. Multi-scale analysis using wavelets will, therefore, detect both micro-level anomalies and macro-level trends to give an insightful understanding of network behavior. A multi-scale residual classifier for anomaly detection that integrates wavelet-based feature extraction with deep learning architectures has been proposed. Residual connections, which are a trademark of modern neural network designs, mitigate the vanishing gradients problem and preserve the representations of features across the layers. This approach therefore illustrates the possibility of fusing wavelet analysis with sophisticated neural network techniques into accurate and overfitting-resilient models useful in real-world applications [14]. The theoretical foundations of wavelets in anomaly detection were further explored by [15], which highlighted the flexibility of wavelet transforms in different network environments and traffic patterns. Their study provides a detailed analysis of how wavelet-based methods can be adapted to various use cases, ranging from detecting distributed denial- of-service (DDoS) attacks to identifying subtle anomalies in normal traffic. This study demonstrates the ability of a hybrid machine learning model combining statistical methods with supervised learning algorithms for anomaly detection. In their approach, they combined wavelet-based preprocessing with the machine learning classifiers to design a hybrid framework that maintains a good balance between the accuracy of detection and efficiency in computation [16]. This methodology shows the continuous relevance of statistical techniques with modern machine learning methods. An autoencoder-based approach to network traffic anomaly detection was introduced, leveraging the ability of autoencoders to learn latent representations of data. By training the model to reconstruct normal traffic patterns, anomalies manifest as deviations from the reconstructed data, enabling precise anomaly detection. This method is particularly effective in identifying subtle, non-obvious anomalies that might elude traditional detection mechanisms [17]. Exploring CNNs for anomaly detection in network traffic, their study highlights the significance of feature hierarchy in deep learning models since CNNs are good at extracting spatial and temporal patterns from complex datasets [18]. By incorporating domain-specific preprocessing techniques, the authors show how deep learning models can be adapted to the unique needs of network traffic analysis. In fact, they proposed a hybrid model, where it uses more than one method to increase the effectiveness of the classification in intrusion detection systems. This ensemble-based approach has made use of several individual models, such as decision trees and neural networks, and combined them in a single framework that learns the strengths of the other to become robust and suitable for all network conditions [19]. This work underlines how ensemble techniques might address some limitations of single-model approaches in handling imbalanced datasets and reducing false positives. The framework for network anomaly detection using isolation forests and autoencoders was introduced. Isolation forests are specifically used in the identification of outliers in high-dimensional datasets, whereas autoencoders are specifically good at capturing latent data structures. Therefore, integrating these methods will yield a framework that is both highly accurate and robust in achieving a promising solution for real-world network environments [20]. Collectively, these works represent important steps toward harnessing wavelet transforms, machine learning, and deep learning to effectively detect anomalies in networks. Wavelet analysis is still a popular choice as a feature extraction tool, because it breaks signals into multi-resolution components and offers an insightful understanding of network traffic patterns. This combination of wavelet-based methods with machine learning models makes for a powerful approach in anomaly detection with high accuracy and low false positives. It is further rich to integrate deep learning techniques into DBNs, LSTMs, and CNNs. The complex temporal and spatial patterns of network traffic may be captured by such models. Such models shine very well when dealing with high-dimensional network data of dynamism and are aptly suitable for modern environments in networks. The introduction of hybrid and ensemble methods is another significant trend because such approaches combine the strengths of multiple techniques to create robust and adaptable frameworks for anomaly detection. Despite these strides, several challenges persist. Real-time applicability poses a critical challenge, as several of the models developed at present cannot handle and process large-scale network traffic within real time. Computational efficiency is another important issue: the complexity of deep learning models often calls for immense computational resources. Finally, the generalizability of these models to diverse network environments and traffic patterns is also an ongoing challenge, for models trained on specific data sets do not perform optimally in other contexts. Table 1 shows the summary of related work for network traffic anomaly detection.

**Table 1:** Summary of related work for network traffic anomaly detection.

Authors	Approach	Features	Contribution	Results
Limthong et al.	Naive Bayes and KNN	Anomaly detection using	Testing of classification	Naive Bayes achieves
		interval based features	algorithms used in the	highest F-measure (0.9)
			study to classify anomalies	while the best performance
			in network based on	of k-NN was 0.75
			interval features	
Aiguo Chen et al.	Hybrid Deep Belief	Generative pretraining to	Improved sequential	Good accuracy and reduced
	Network(DBN)-LSTM	improve performance of	anomaly detection in	time computation in the
	Network	LSTM	dynamic network traffic	identification of anomalies
				in network behavior along
				with quick model update
				capability.
M.Salagean et al.	Discrete Wavelet Transform	Frequency decomposition	Focused on granular	Has achieved high
		of traffic data	decompositions of traffic	precision in detection of
			signals to detect network	subtle anomalies in real
			anomalies with low false	deployments
			positives	
Novakov et al.	Wavelet Transform with	PCA for dimensionality	Combined PCA to reduce	Improved the
	PCA	reduction, combined with	computational complexity	computational efficiency
		wavelets	while keeping significant	and scalability to the
			features for anomaly	high-dimensional network
			detection	traffic for real-time
				anomaly detection
Jiang et al.	Continuous Wavelet	Multi-scale analysis for	Application of continuous	Scalable and accurate
	Transform and PCA	detecting large-scale as	wavelet transforms to	anomaly detection in broad
		well as fine-grained	detect both coarse and	network environments
		anomalies	fine-grained anomalies in	
			high-speed networks	
Xueyuan Duan et al.	Wavelet Transform and	Feature extraction using	Combination of wavelet	Models are made more
	Stacked automatic encoder	Wavelet-based with residual	analysis with Deep learning	robust so that correct and
		Deep Learning classifiers	for vanishing gradient	robust anomalies are
			issues	obtained in dynamic
				network traffic
Kaur et al.	Wavelet Transforms	General wavelet transforms	Wavelet's usage in	Enabled large-scale usage
		for different network	detection of DDoS attacks	of this anomaly detection
		environments	as well as fine-grained	scheme, that encompassed
			anomalies within normal	the wide spectrum of
			traffic	anomalies
Taeshik Shon et al.	Self Organized Feature	Statistical methods	Integration of wavelet	Statistical method-based
	Map (SOFM), Passive	combined with machine	preprocessing with	machine learning to
	TCP/IP Fingerprinting,	learning classifiers	statistical and machine	enhance anomaly detection
	Genetic Algorithm and		learning methods for	while minimizing false
	Temporal Flow		accuracy and efficiency	positives
K.Korniszuk et al.	Autoencoders	Autoencoders to learn	Application of	Achieves efficient anomaly
		latent features	autoencoders focusing on	detection with minimal
			anomaly detection by	supervision such that false
			performing comparisons of	positives are removed.
			reconstructed traffic	
			patterns	
K. Sharma et al.	Convolutional Neural	CNNs to extract spatial and	Applied CNNs for	Achieved 95 percent
	Networks	temporal patterns	hierarchical feature and	accuracy for classifying
			pattern extraction from	network anomalies with
			network traffic for anomaly	structured traffic patterns
			detection	
Dutta et al.	Classical Autoencoder and	Hybrid classical	Hybrid framework	Enhanced performance by
	Deep Neural Networks	autoencoder and deep	improved accuracy and	reduced false positives
		neural network framework	better classification	
		to capture relevant features		
		of complex data		
M.G. et al.	Combination of Isolation	Hybrid framework helps	Leverage Isolation Forests	Resulted in High Accuracy
	Forests and Autoencoders	learn complex data	in Outlier Detection and	in High-Dimensional Data
		representations in high	Autoencoders for Latent	
		dimensions	Features Learning	

## Method details

A. About the Dataset

**Fig. 1:**
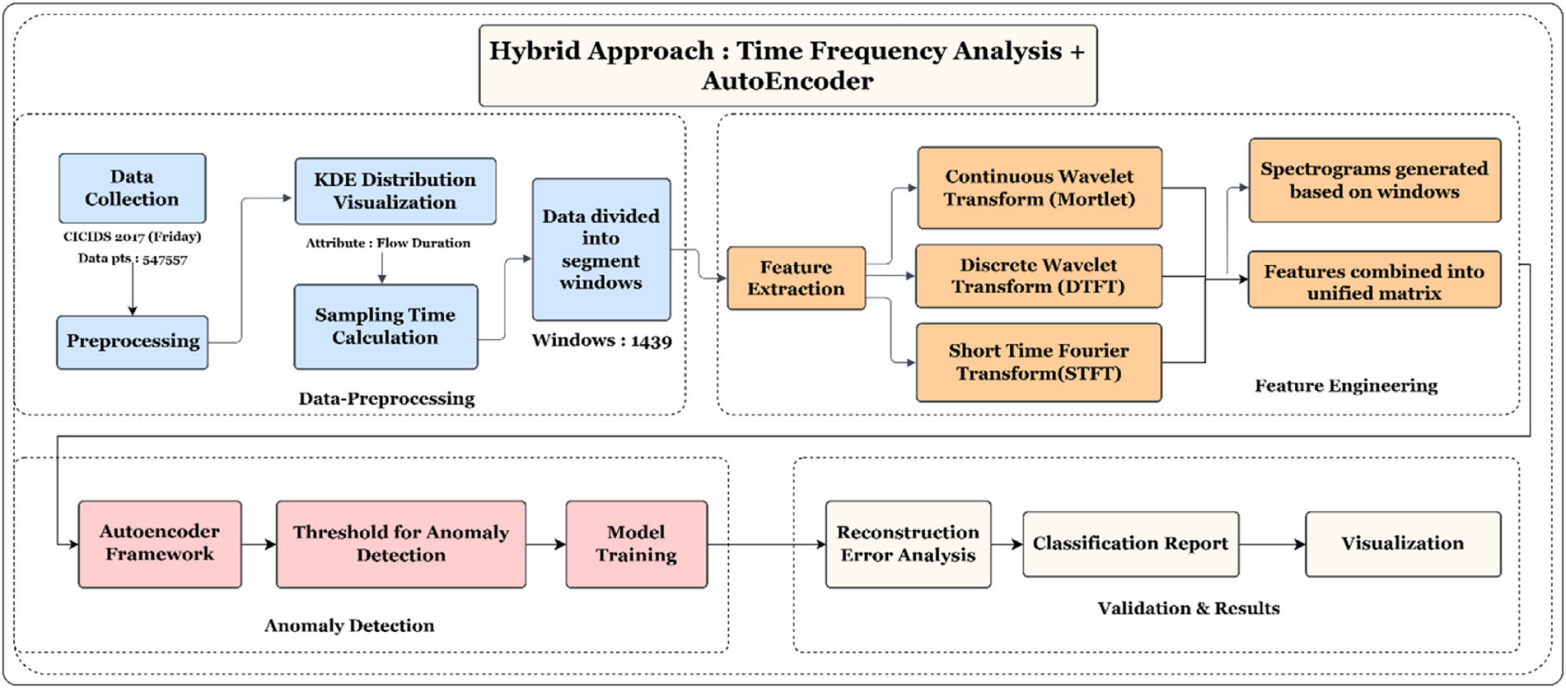
Methodology for network traffic anomaly detection.

The dataset applied in this research is from the CICIDS 2017 collection [21]. Capture network traffic data, including various characteristics such as packet flow duration, protocol types, and other network behavior characteristics. The dataset contains about 5,47,557 data points. The dataset is loaded and prepared for analysis. Missing values introduce noise or bias in data and thus are dropped. The study focuses on the Flow Duration feature that represents the time a connection is active. The feature has been essential for identifying anomalies like long-lasting connections associated with DDoS attacks or irregular network behaviors [22]. The detailed methodology is shown in Fig. 1.

B. Kernel Distribution Estimation of Flow Duration

KDE is a non-parametric technique that estimates the probability density function of a continuous variable, thus providing a smooth representation of the data distribution. From the graph, it can be seen that the data is highly right-skewed, meaning most of the values of the duration are concentrated around the smaller ranges, close to zero [11]. The density curve is showing a sharp peak near the origin, indicating that most of the flows have very short durations. This is normal in network or flow-based data, where the smaller durations dominate because of short interactions or events [23].

**Fig. 2:**
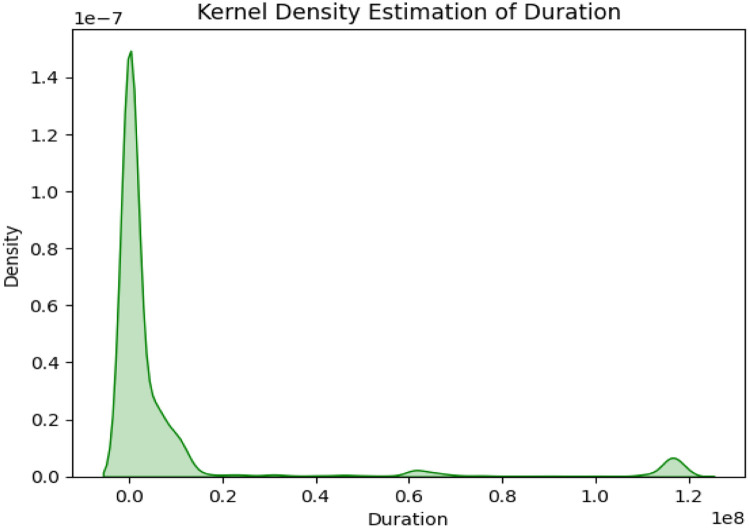
KDE of flow duration.

From Fig. 2 the green color and the filled area below the curve make the shape of the distribution visually intuitive. The density drops off very sharply as the duration increases, approaching near- zero values as it moves toward longer durations. There are a few small bumps in the right tail of the curve that indicate the presence of outliers or less frequent events with unusually long durations. However, most of the density is concentrated within the range at the beginning, which illustrates the skewness. The smooth curve of the KDE allows for a better view of the general shape of the distribution than a histogram does, especially when data is continuous.

C. Continuous Wavelet Transform (CWT)

The Continuous Wavelet Transform (CWT) is an advanced signal processing method that can be used for time-frequency analysis. This allows the representation of characteristics of a signal with respect to different scales, or resolutions. In contrast to traditional Fourier transforms, which provide a global description of a signal's frequency, the CWT presents both a view of frequency as well as time domains of the signal. Such characteristics are very useful for time-varying, and in particular, for the analysis of non-stationary or transient signals [24]. This research utilizes CWT to decompose the signal into time-frequency representation using the Morlet wavelet. The Morlet wavelet is generally adopted in most time-frequency analyses since it offers an optimal compromise between both time and frequency localizations. It is defined by being a complex sinusoid that is modulated by a Gaussian window, providing thus a good compromise between high frequencies localization in time and localization at low frequencies in frequency [25].

The general equation for the CWT is given by following Eq. (1)

(1) $$W\left(a,b\right)=\int_{-\infty }^{\infty }f\left(t\right)\;\psi *\left(\frac{t-b}{a}\right)\;dt$$

where W(a,b) is the wavelet coefficient, f (t) is the signal to be analyzed, ψ∗ is the complex conjugate of the wavelet function, a determines width of wavelet, and b determines shift of wavelet in time [26].

In this study, the CWT is used to apply the transform to the signal in order to examine its frequency content at different scales. The signal is decomposed into a time-frequency representation, here the signal is split into smaller-sized windows, and it is convolved with a series of wavelets at different scales and positions. The coefficients obtained are the amplitude of the wavelet at each scale and time, which can be visualized in a time-frequency plot or spectrogram. The CWT catches localized features of the signal and monitors its evolution of how the frequency content is coming over time. This lets us determine transient behaviors while tracking the changes in variation of the frequency components on localized time-frequency spectrograms. This approach is very effective in the detection of patterns or anomalies in signals with time-varying behavior.

D. Discrete Time Fourier Transform (DTFT)

The Discrete-Time Fourier Transform is used to analyze the frequency content of discrete-time signals. This is in contrast to the Discrete Fourier Transform that is calculated for a number of data points. For an infinite signal, it gives a continuous frequency spectrum. It is very helpful in determining how a signal is composed of different frequency components and is thus of great use in analyzing periodic and sampled signals [27].

This study utilizes DTFT on signal segments or windows to analyze how the signal's frequency content varies over time. The DTFT is applied to each window, that allows us to capture crucial aspects regarding the spectral features of the signal, such as dominant frequencies and bandwidths, at any given time point [28].

The DTFT is mathematically defined by the following Eq. (2):

(2) $$X\left(e^{j\omega }\right)=\sum_{-\infty }^{\infty }x\left[n\right]e^{-j\omega n}$$

where $X(e^{j\omega }$) is the DTFT of signal x[n] is discrete time signal, ω represents the angular frequency, and j is the imaginary unit [29]. This approach avoids discretizing the frequency spectrum into fixed bins and gives transient behavior along with significant frequency components at other instances of time which helps get a more detailed analysis of signal's frequency content.

E. Short-Time Fourier Transform (STFT)

The Short-Time Fourier Transform is a technique that divides a signal into overlapping windows and then applies the Fourier transform to each window in order to analyze the frequency content over time [30]. Unlike the Fourier Transform, which gives a global frequency representation, the STFT provides a time-frequency representation, making it useful for analyzing non-stationary signals whose frequency characteristics change over time. The STFT is used in many applications, including speech analysis, audio signal processing, and time-varying spectral analysis. In this study, the STFT is applied on a segmented window of the signal to see how the frequency content varies in each window. With the Fourier transform on each window, it is possible to obtain a time-frequency representation that would reveal the variation of the frequency components in the signal over time. It is specifically useful for transient behaviors and time-varying frequencies of signals that could not be completely captured in a single global frequency spectrum [31]. The STFT can be mathematically defined as using Eq. (3):

(3) $$X\left(\omega ,\tau \right)=\int_{-\infty }^{\infty }x\left(t\right)\;w\left(t-\tau \right)e^{-j\omega t}\;dt\;$$

where X(ω, τ) represents the STFT of the signal x(t) is the time index, w(t − τ) is the window function centered at time τ, and e−jωt is the Fourier kernel [32]. The STFT in practice is actually a computation of the Fourier transform over small overlapping segments of the signal. It allows us to track the evolution of frequency components across time. It is best suited for signals that have time-varying frequency characteristics. The reason is that it gives localized frequency information evolving with time, and it makes possible a better understanding of transient features and spectral changes in the signal.

F. Autoencoder

An autoencoder is a type of unsupervised neural network: it learns an efficient compact representation of the input, usually by minimizing the reconstruction error between the original and reconstructed input [33]. An autoencoder architecture is comprised of two main parts: the encoder and the decoder.

Encoder: Encoder compresses input data to a lower dimensional representation. This compressed version is referred to as latent space or bottleneck. Compressed form is expected to contain all the essential information and discard the irrelevant once. Encoder then progressively decreases the dimensionality of input through multiple layers so that the model gets focused on the main attributes of the normal data.

Decoder: The decoder tries to reconstruct the original input data from the compressed representation produced by the encoder. It expands the lower-dimensional latent space back to the original input dimension, using the learned features to recreate the data as closely as possible. In case the data is somewhat similar to the normal patterns the model was trained on, the reconstruction will be accurate with minimal error.

In the context of anomaly detection, the autoencoder is trained to have learned normal data well for effective reconstruction. However, while being presented with anomalous data, the autoencoder can only reconstruct it poorly, due to the fact that these patterns in the anomaly did not exist in its normal training data. In contrast, this leads to more reconstruction errors for anomalies on data points [34]. Continuous Wavelet Transform (CWT), Discrete-Time Fourier Transform (DTFT), and Short-Time Fourier Transform (STFT) transformations are used to produce the input features to increase the ability of the autoencoder in anomaly detection. This will capture the time-frequency characteristics of the data that would provide richer information the autoencoder can utilize for learning better representations [35]. Outputs of these transformations are combined and used as input features of the autoencoder. In this hybrid approach, it allows the autoencoder to learn a more comprehensive representation of normal patterns in data so that it can be sensitive to anomalies. Then anomalies are detected by higher reconstruction errors, which occur whenever the model is encountering data that significantly deviates from the learned normal patterns.

## Method validation

### Continuous wavelet transform

**Fig. 3:**
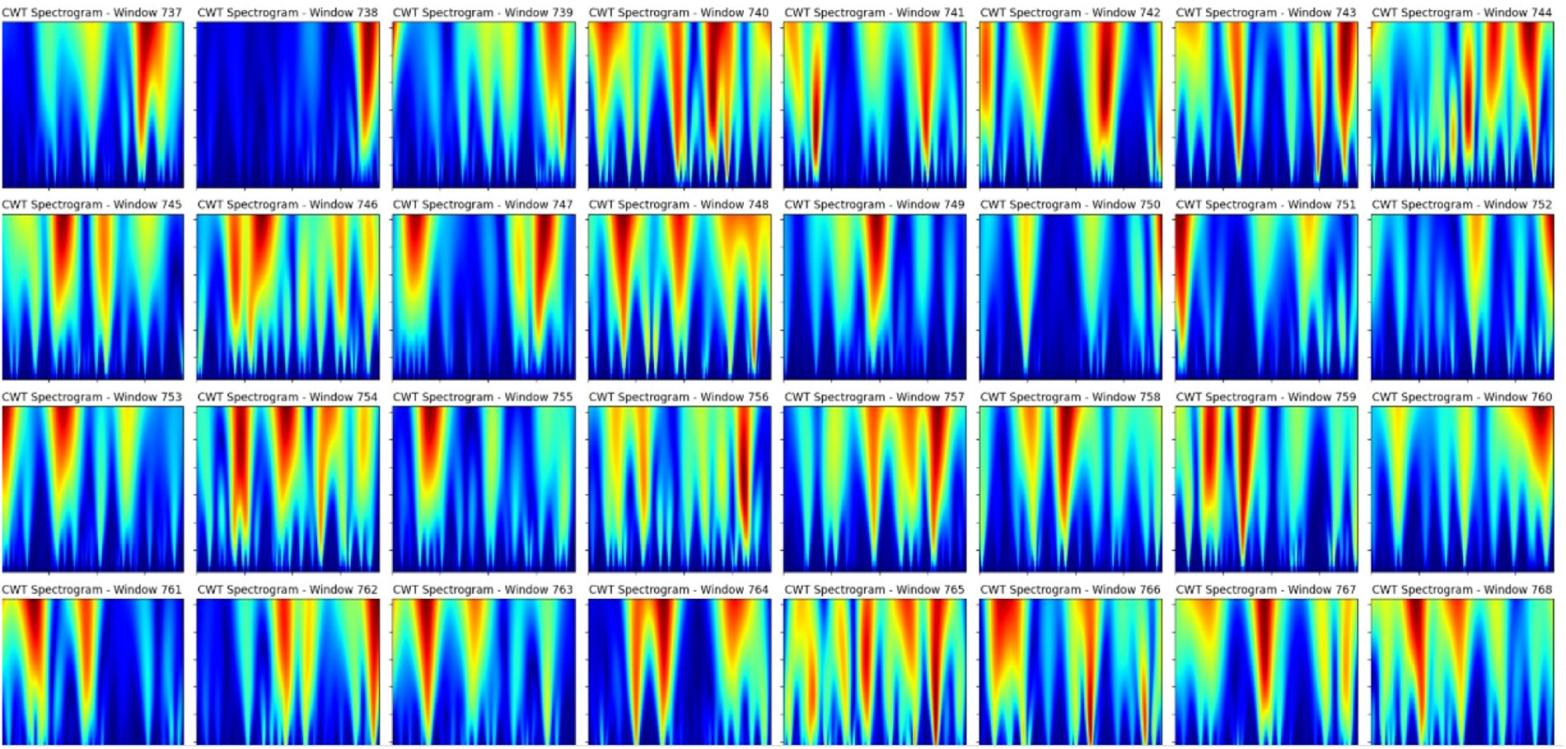
CWT plot of 1439 windows.

The continuous wavelet transform (CWT) is a powerful time-frequency representation that allows for further analysis of signal behavior at any point in time. Fig. 3 shows that dynamic changes in the signals are observed across multiple windows of CWT spectrograms. Among the 1439 CWT spectrograms, there were distinct energy distributions with red-yellow regions marking higher power concentrations, especially on low frequencies. This again suggests that the main signal features are stationary or periodic.

However, at some time windows, the blue regions of intermittent high- frequency components indicate transient events or anomalies in the signal. Anomaly detection is best achieved by CWT because it localizes signal changes both in time and frequency domains. The red-yellow regions of time-localized energy spikes may indicate an abrupt event, noise burst, or fault occurrence at irregular time windows. Discontinuities in frequency distributions between successive windows can expose anomalies in normal signal operation.

The fine time resolution of CWT enables the detection of short-duration anomalies that traditional methods, such as FFT, may miss. Moreover, multiscale decomposition identifies coarse trends (low-frequency signals) and fine details (high- frequency noise), which provides a holistic view of signal behavior.

### Discrete-time Fourier transform (DTFT)

**Fig. 4:**
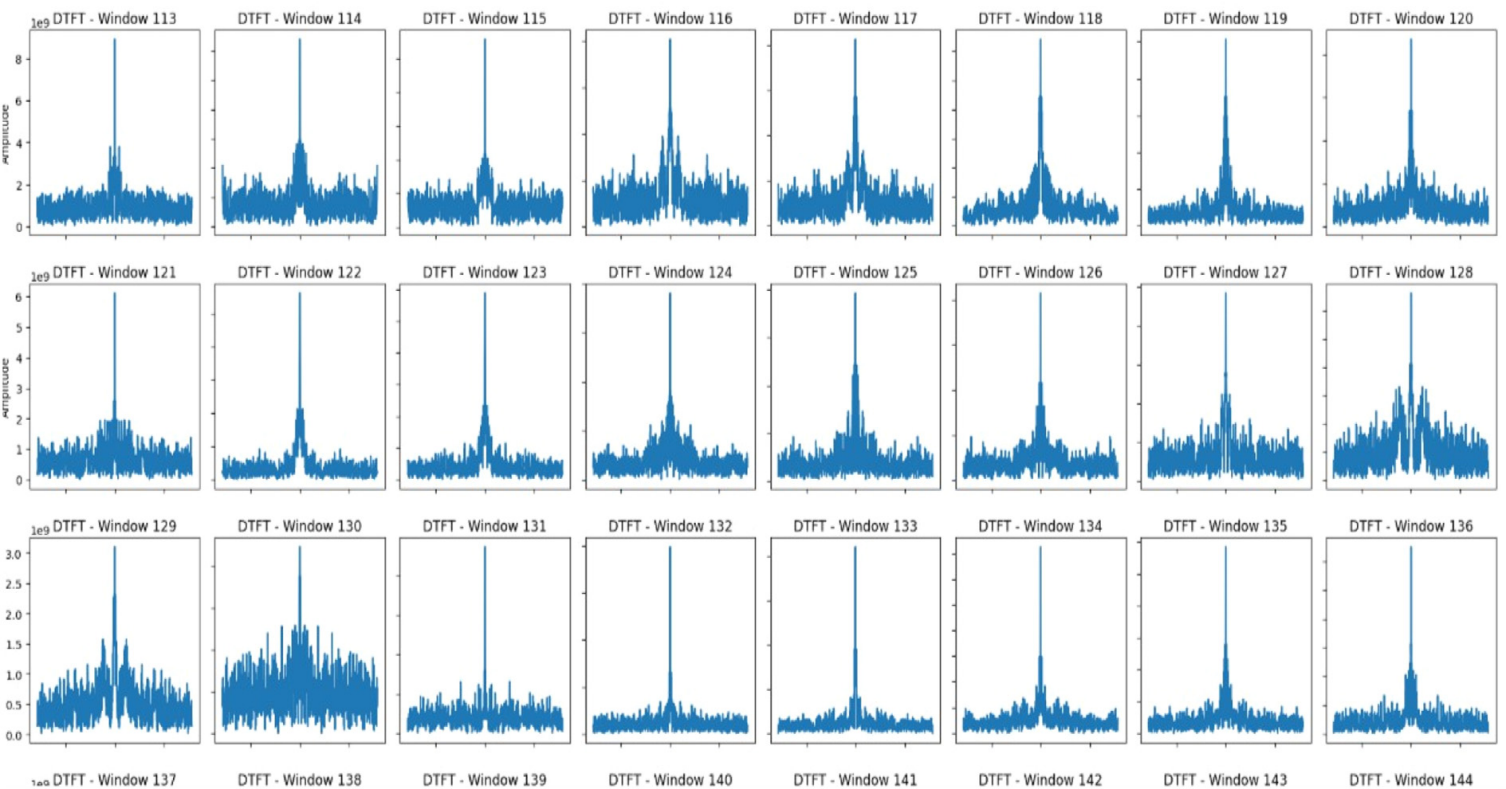
DTFT plot of 1439 windows.

Most of the windows show clear periodic behavior and a well-defined dominant frequency component. This is evidenced by sharp peaks centered around a certain frequency, supported by relatively low-amplitude side lobes. In such cases, spectral stability is obvious, with negligible variations or disruptions in such windows. However, one prominent anomaly across all windows is a dark vertical line, indicating perhaps a synchronized frequency spike happening to all time windows simultaneously at the same instant in time. In Fig. 4, the DTFT analysis reflects several anomalies in the frequency axis, indicating possible signal peculiarities. Most windows are associated with stable spectral characteristics accompanied by consistent dominant frequencies whereas localized anomalies are visible.

There are some windows with highly increased amplitude spikes at the dominant frequency, which could indicate transient energy pulses or signal interference. Windows 35, 99, 125 and 178 have sharp amplitude peaks that differ considerably from adjacent windows. Pruned side-lobe noise is detected in certain windows, which implies interference or more extensive spectral spread. Windows 107 and 139 show wider and noisier side-lobe energy. Window 339 and 363 have uneven amplitude distributions at the location of the major frequency.

Small deviation in frequency positioning is noticed in Windows 128 and 141 as peaks appear slightly skewed or flattened. A vertical dark line across all windows indicates a global interference event or a transient burst of energy across the system. Window 130 indicates irregular energy dispersion around the prominent peak, and Window 143 shows a broader spectrum with less defined peaks.

### Short-time Fourier transform (STFT)

The STFT spectrograms give a visual representation of the signal's frequency content over time, where color intensity corresponds to the energy or magnitude of the frequency components. The spectrograms range in blue and cyan shades with darker blues indicating greater intensity in some areas while the others are lighter shades. Variations in the color intensity across the spectrograms may indicate changes in energy distribution within the frequency bands of the signal over time. Areas with a sudden or abrupt coloration change might imply that the anomalies include transients, frequency jumps, or drastic changes in signal properties.

**Fig. 5:**
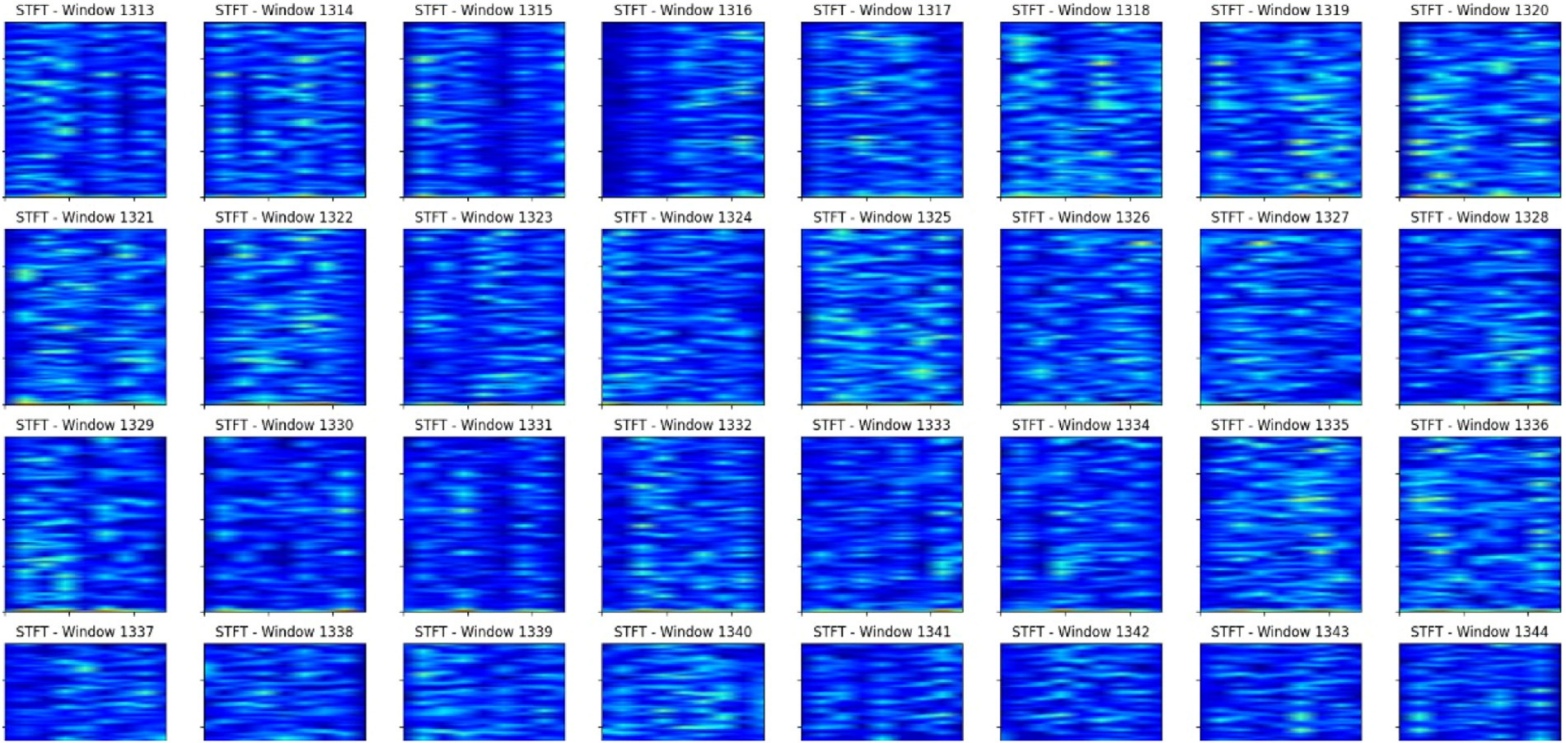
STFT plot of 1439 windows.

From the spectrograms in Fig. 5, energy distributions can be visually sensed by vertical patterns or "stripes” on the image. Some frequency bands might exhibit stronger activity or energy concentration and, therefore, are linked to certain signal components or physical processes. Some anomalies could appear as a local distortion or a violation of the expected pattern of frequency bands, for instance, by the presence of some unexpected frequency components or by the disappearance of the dominating frequency bands.

The horizontal axis of the spectrograms represents the time domain, allowing us to observe how the signal's frequency content evolves over time. Consistent or periodic patterns in the spectrograms may indicate regular or cyclic behavior in the underlying signal, while sudden changes or irregularities in these patterns could signify the presence of anomalies.

### Reconstruction error

Reconstruction error refers to the difference between the original data and the reconstructed data of an autoencoder model, which is a kind of neural network applied in the process of dimensionality reduction and anomaly detection.

**Fig. 6:**
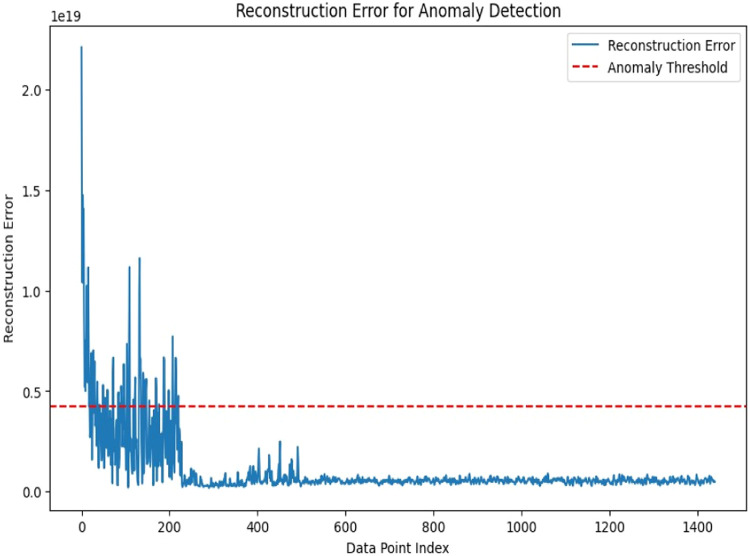
Reconstruction error.

Autoencoders are trained to learn a compressed representation of the input data and then reconstruct the original input from this compressed representation. A large reconstruction error suggests that the point is significantly deviating from the normal patterns learned by the autoencoder. The anomaly threshold, or the red line, is a predefined value that demarcates normal and anomalous data points. Where the reconstruction error surpasses the anomaly threshold, the possibility of an anomaly is inferred in the data. This visualization of the anomaly detection process can pick out the data points, which don't fit within the model. The graph of the reconstruction error in Fig. 6 shows how well the autoencoder is at picking up underlying patterns of the data. Regions with consistently low reconstruction error indicate that the autoencoder has learned the normal data distribution well, while regions with high reconstruction error highlight where the autoencoder is having difficulty reconstructing the data.

## Detected anomalies

**Fig. 7:**
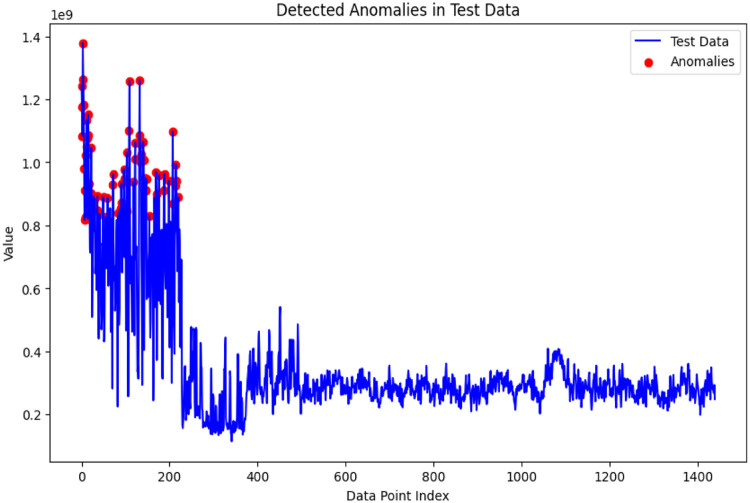
Detected anomalies.

The Fig. 7 demonstrates a plot of detected anomalies that help visualize the process of anomaly detection. We have detected a total of 72 anomalies and achieved 95 percent accuracy. The red dots at the plot are identified to be data points belonging to anomalies. Analyzing how these anomalies are spatially located and distributed offers insightful information regarding the characteristics or nature of deviations of abnormal data patterns.

## Conclusion

This paper proposed a hybrid approach that combines CWT, DTFT, and STFT with autoencoders for network traffic anomaly detection. Integrating time-frequency analysis with machine learning enables the model to detect even subtle, short-duration anomalies that conventional methods often miss. The model is robust and scalable for real-time applications, achieving 95% detection accuracy and identifying 72 anomalies. These results demonstrate the feasibility of deploying this approach in practical cybersecurity applications.

## Limitations

None.

## Ethics statements

Not applicable.

## CRediT authorship contribution statement

**Ruchira Purohit:** Conceptualization, Methodology, Formal analysis, Data curation, Writing – original draft. **Satish Kumar:** Conceptualization, Resources, Supervision, Funding acquisition. **Sameer Sayyad:** Conceptualization, Methodology, Writing – review & editing, Resources. **Ketan Kotecha:** Resources, Supervision, Funding acquisition.

## Declaration of competing interest

The authors declare that they have no known competing financial interests or personal relationships that could have appeared to influence the work reported in this paper.

## Data Availability

Data will be made available on request.
